# Case Report: *Anaerococcus vaginalis* spondylodiscitis diagnosed and cured based on nanopore targeted sequencing

**DOI:** 10.3389/fmed.2025.1645609

**Published:** 2025-07-30

**Authors:** Bing Zhou, Youfeng Guo, Beiduo Shen, Ziqi Zhu, Bin Yu, Gen Li, Ming Zong, Tao Hu, Desheng Wu

**Affiliations:** ^1^Department of Spine Surgery, Shanghai East Hospital, School of Medicine, Tongji University, Shanghai, China; ^2^Department of Orthopedics, Gaoyou People’s Hospital, Gaoyou, China; ^3^Department of Clinical Laboratory, Shanghai East Hospital, School of Medicine, Tongji University, Shanghai, China; ^4^Institute for Advanced Study, Tongji University, Shanghai, China

**Keywords:** *Anaerococcus vaginalis*, spondylodiscitis, nanopore targeted sequencing, diagnosis, treatment

## Abstract

Spondylodiscitis caused by *Anaerococcus vaginalis* has not been reported yet, making it worthy of further exploration. We closely monitored a patient who experienced worsened low back pain for 1 month and was unable to walk due to severe pain for 1 week. Through thorough examinations, the patient was diagnosed with lumbar disc herniation and a suspected case of spondylodiscitis, necessitating posterior lumbar surgery. All culture results returned negative. However, nanopore targeted sequencing (NTS) identified the presence of *Anaerococcus vaginalis*. Subsequent administration of appropriate antibiotics resulted in a favorable clinical outcome for the patient.

## 1 Introduction

The incidence of spondylodiscitis is very rare, ranging from 4 to 24 in a million each year (1). Spondylodiscitis is frequently attributed to aerobic pathogen infections, while rarely anaerobic pathogens (2). The diagnosis of spondylodiscitis is frequently delayed or overlooked due to its insidious onset and the nonspecific nature of its signs and symptoms, particularly when caused by opportunistic pathogens (3). *Anaerococcus vaginalis* is an anaerobic, Gram-positive, opportunistic pathogen (4). Spondylodiscitis attributed to *Anaerococcus vaginalis* had not been previously documented in the literature. We identified this condition using nanopore targeted sequencing (NTS) and are the first to report such a case.

## 2 Case report

**Figure 1:**
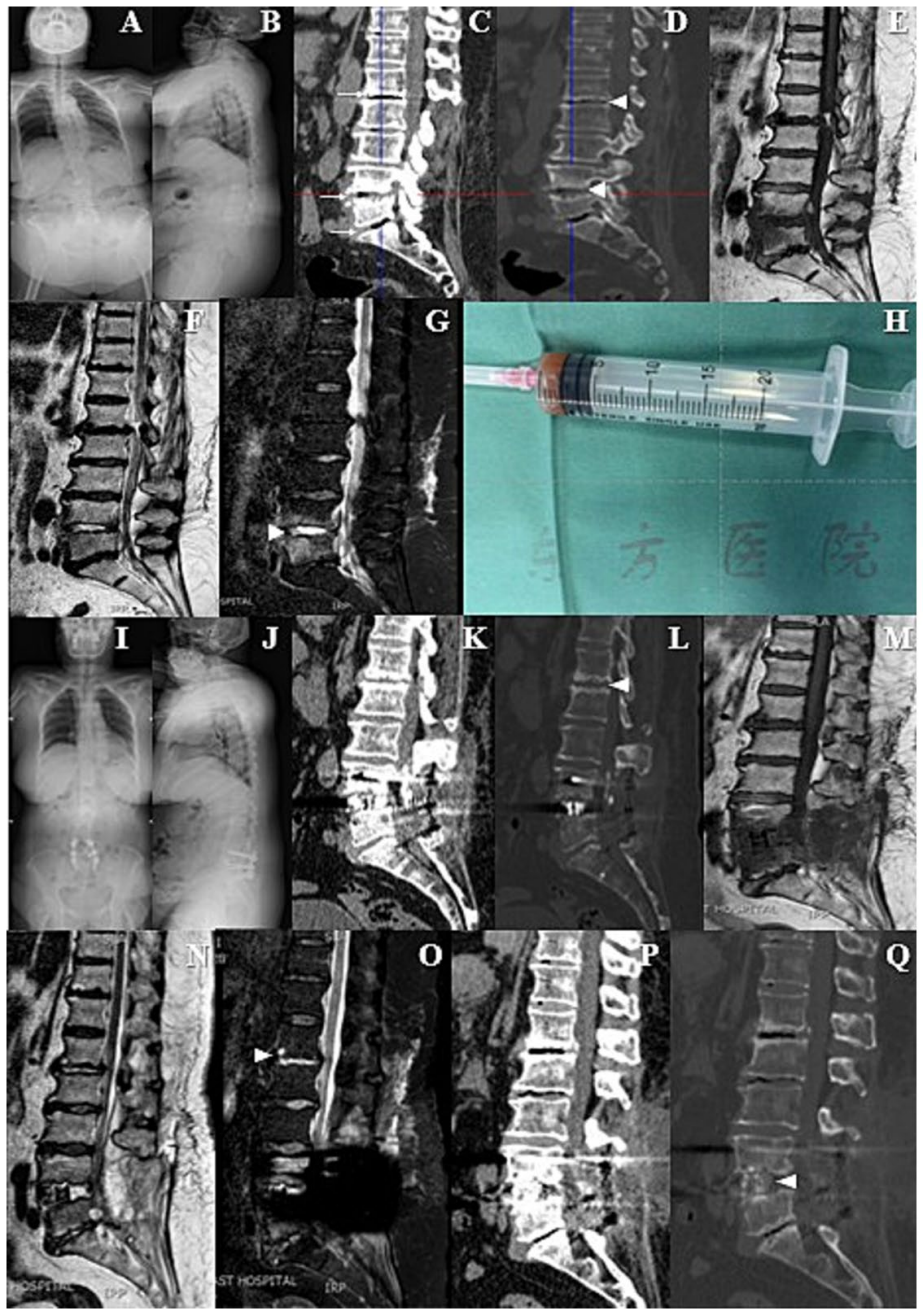
**(A,B)** Preoperative X-ray films showed scoliosis and degenerative changes in the spine. **(C,D)** Preoperative computed tomography (CT) revealed obvious linear gas density shadows in the L1/2, L4/5 and L5/S1 intervertebral discs. In addition, the upper and lower endplates of L1/2 and L4/5 vertebrae showed characteristic “worm-eaten” destructive changes. **(E–G)** Preoperative magnetic resonance imaging (MRI) demonstrated significantly enhanced signals in the L4/5 intervertebral disc. **(H)** During the operation, obvious destruction of the L4/5 intervertebral disc was observed, accompanied by reddish-brown fluid exudation, which was aspirated for subsequent bacterial culture, fungal culture and gene sequencing. **(I–L)** Pre-discharge X-ray and CT films indicated thorough decompression and satisfactory internal fixation. **(M–O)** Follow-up MRI showed that the high signal intensity of L4/5 intervertebral disc had decreased, while the signal brightness of L1/2 intervertebral disc had increased. **(P,Q)** Follow-up CT showed no significant difference compared with the pre-discharge CT, except for new bone formation in the L4/5 intervertebral space that did not meet the fusion criteria.

A 71-year-old female patient was admitted to our institution, arriving in a wheelchair. She reported a one-month history of exacerbated low back pain and had been unable to ambulate for one week due to the rapid onset of pain. Physical examination indicated a Visual Analogue Scale (VAS) score of 8 for lumbar pain, with notable percussion tenderness over the L4 to L5 vertebrae and restricted lumbar flexion and extension. Muscle strength testing demonstrated a 3/5 strength in all muscle groups of both lower limbs. Sensory examination revealed decreased sensation in both legs, while body temperature remained within normal limits. Laboratory investigations showed a slight elevation in C-reactive protein (CRP) levels (35.22 mg/L) and erythrocyte sedimentation rate (ESR) (47 mm/h), whereas the white blood cell (WBC) count and neutrophil count were within normal ranges. Routine blood and urine cultures were negative. Radiographs revealed scoliosis and degenerative changes in the spine (Figures 1A,B). Computerized tomography (CT) showed the obvious linear gas density shadows occurred in the discs of L1/2, L4/5 and L5/S1. Furthermore, the superior and inferior endplates of L1/2 and L4/5 exhibited a distinctive “worm-eaten” pattern of destruction (Figures 1C,D). The magnetic resonance imaging (MRI) demonstrated a highlighted signal in the intervertebral disc of L4/5 (Figures 1E–G).

The patient received a diagnosis of lumbar disc herniation, along with a suspected spondylodiscitis. She underwent posterior lumbar interbody fusion (PLIF) at L4–L5 and posterolateral lumbar fusion (PLF) at L5–S1. Intraoperatively, a noticeable erosion of the L4/5 intervertebral disc was observed, accompanied by reddish-brown fluid exudate. This fluid was aspirated for subsequent bacterial and fungal cultures, as well as gene sequencing (Figure 1H). Simultaneously, the excised disc tissue was sent for pathological examination. The surgical procedure was performed without any complications or unforeseen events.

**Figure 2:**
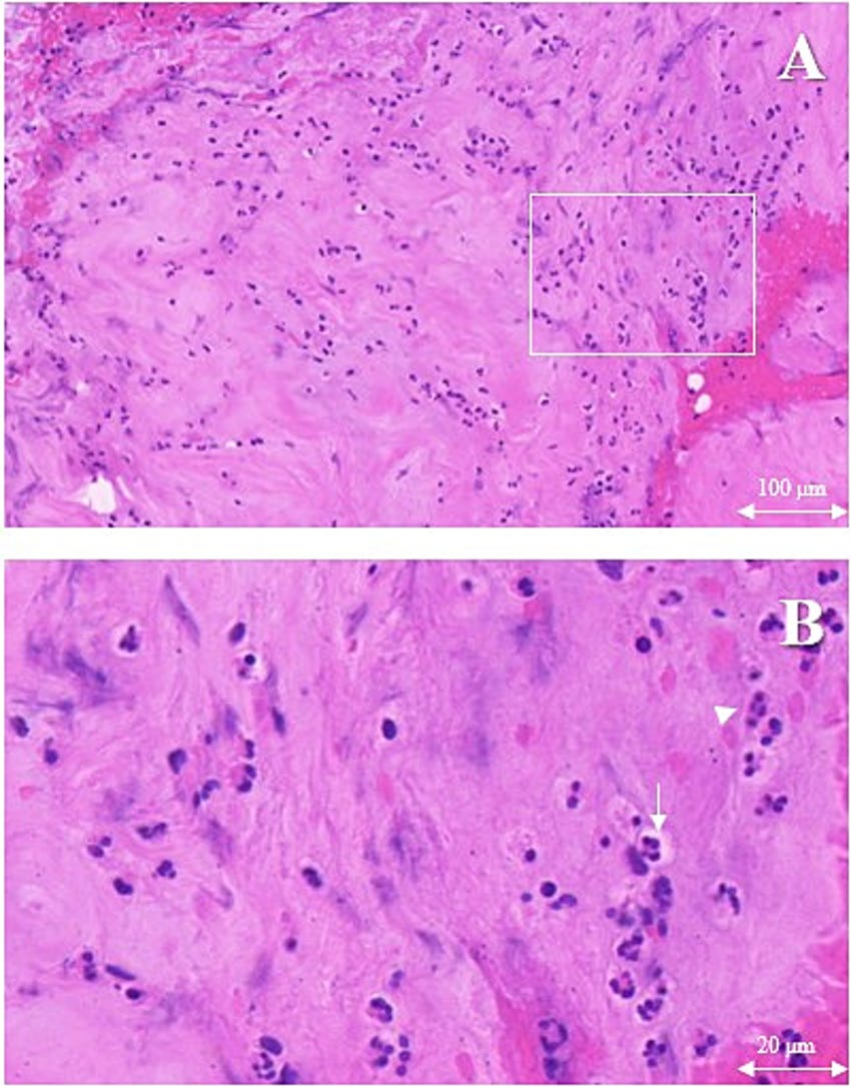
The erosional disc tissue with hematoxylin and eosin. Chondrocytes (white arrows) accompanied by inflammatory cells (white triangle) infiltration. **(A)** Magnification ×10, scale bar 100 μm, **(B)** Magnification ×40, scale bar 20 μm.

**Figure 3:**
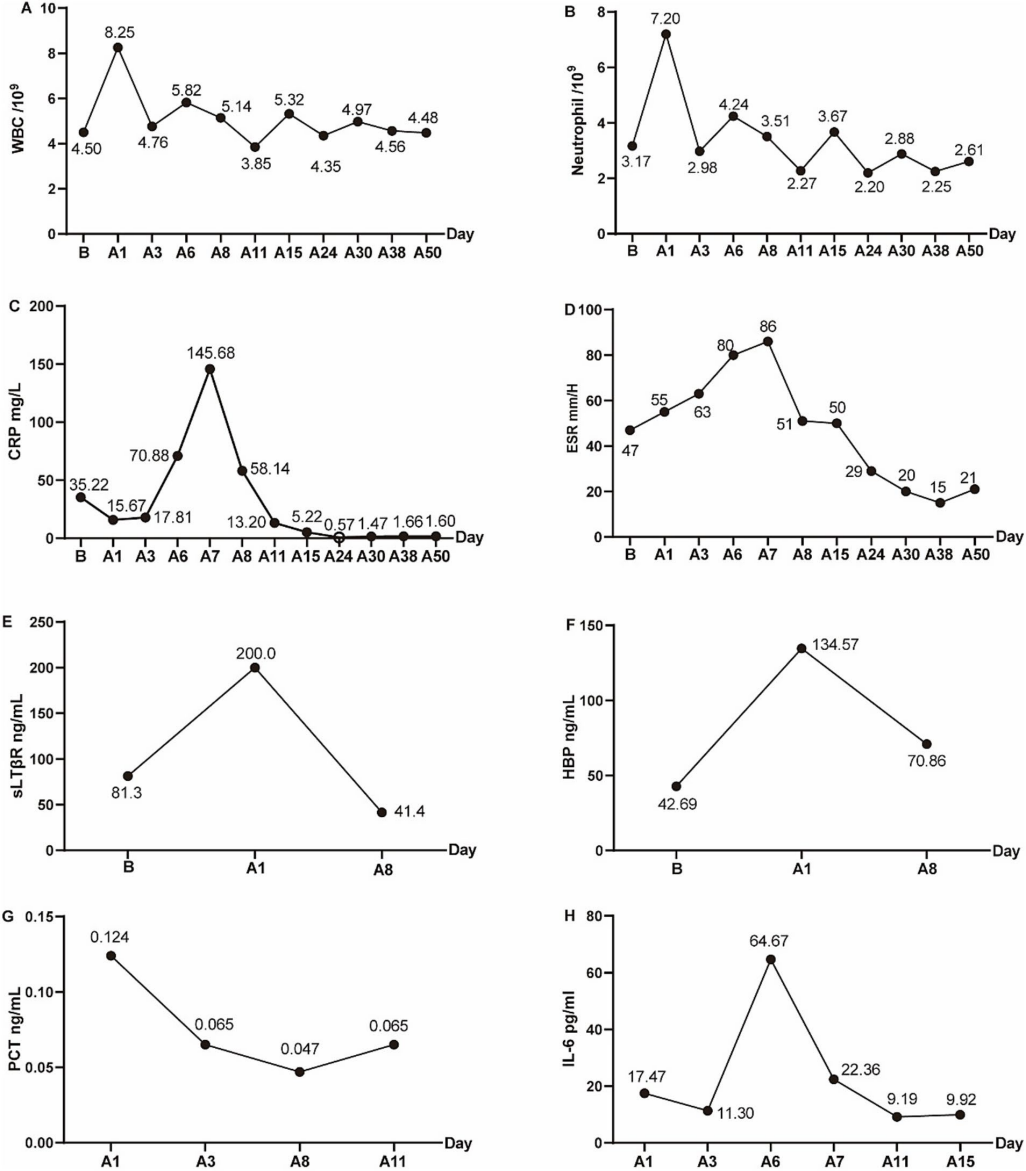
The peripheral-blood inflammatory markers preoperatively and postoperatively. **(A)** White blood cell (WBC), **(B)** Neutrophil, **(C)** C-reactive protein (CRP), **(D)** Erythrocyte sedimentation rate (ESR), **(E)** sLTβR, **(F)** HBP, **(G)** PCT, **(H)** IL-6. “B” indicated before operation, and “A” indicated after operation. A1 indicated 1 day after the operation, similarly, A3, A5, A6, A7, A8, A11, A15, A24, A30, A38, A50 indicated 3, 5, 6, 7, 8, 11, 15, 24, 30, 38, 50 days after operation.

On the first postoperative day, the patient was initiated on empirical antimicrobial therapy, comprising intravenous Vancomycin at a dosage of 500 mg every 12 h, in conjunction with intravenous Levofloxacin at a daily dosage of 0.5 g. The pathological report revealed degenerative changes and localized necrosis accompanied by inflammatory cell infiltration in the examined fibrocartilaginous tissue specimen (Figures 2A,B). As anticipated, the cultures conducted on the extracted fluid consistently yielded negative results. Nonetheless, NTS identified the presence of *Anaerococcus vaginalis*. Consequently, Levofloxacin was substituted with intravenous Metronidazole, administered at a dosage of 0.5 g every 12 h, to which the organism demonstrated susceptibility. The patient’s inflammatory markers showed varying degrees of elevation postoperatively, but the majority of inflammatory markers had roughly returned to normal levels following a two-week course of continued intravenous antibiotic therapy (Figures 3A–H). Furthermore, during the follow-up, the inflammatory markers WBC, Neu, ESR, and CRP all remained consistently within a stable and normal range (Figures 3A–D).

Fifteen days postoperatively, the patient had attained independence in walking and was smoothly discharged with a one-month course of oral antibiotics (metronidazole 0.2 g/tid and celecoxib 0.2 g/bid). The VAS score for lumbar pain had notably decreased from 8 to 2. The pre-discharge radiographs suggested thorough decompression and satisfying internal fixation (Figures 1I–L). In addition, the MRI revealed that a highlighted signal intensity of the L4/5 intervertebral disc had decreased, whereas the L1/2 intervertebral disc exhibited increased brightness (Figures 1M–O). The CT bone tissue image also revealed the heightened density in the superior and inferior endplate regions of the L1/2 intervertebral disc (Figure 1L). The follow-up CT showed no significant deviation from the pre-discharge CT, except for new bone formation at the L4/5 intervertebral space but not meet the fusion criteria (Figures 1P,Q).

## 3 Discussion

The presentations of spondylodiscitis were not highly specific, especially opportunistic pathogens, making it challenging to definitive diagnosis (5). The pivotal factor in the diagnosis of spondylodiscitis was identifying pathogens. In the absence of conclusive evidence, empirical antimicrobial therapy continues to be the predominant strategy, which may result in suboptimal treatment outcomes, increased antimicrobial resistance, and a heightened risk of adverse effects and mortality (6, 7). Microbial culture was insensitive and prolonged (8). The PCR method exhibited limited coverage and inefficiency (6). The metagenomic next-generation sequencing (mNGS) entailed high costs, lengthy detection cycles, complex bioinformatics processing, and challenges in achieving standardization (9). NTS offered unique advantages for the rapid diagnosis of infectious diseases, including speediness, economy, and accuracy, overcoming the limitations of culture, PCR, and mNGS (9).

Although multiple culture results were consistently negative in this article, we successfully identified the presence of *Anaerococcus vaginalis* through NTS. NTS has been also documented to possess distinct advantages in the identification of pathogenic bacteria in cases of lung infections and serious infections, significantly contributing to guiding anti-infection treatment and mitigating mortality (7, 9). The intervertebral disc was an inherently sterile and avascular tissue, which contributed to the distinct and localized nature of intervertebral disc infection (10). Consequently, diagnosing spondylodiscitis presents significant challenges, particularly when it is caused by low-virulence opportunistic pathogens that may only be conclusively identified through gene sequencing techniques such as NGS. Nevertheless, the feasibility of employing NGS as a routine or standard diagnostic approach for spondylodiscitis warrants further exploration.

While NTS proved instrumental in identifying *Anaerococcus vaginalis* in this first reported case of *A. vaginalis* spondylodiscitis, the biological basis of this unusual infection warrants discussion. *A. vaginalis* is a Gram-positive, anaerobic coccus typically found in vaginal microbiota but increasingly recognized as an opportunistic pathogen in polymicrobial infections. Its successful establishment in the intervertebral disc likely reflects both pathogen-specific adaptations and host factors: the avascular, hypoxic disc microenvironment provides an ideal niche for anaerobic growth, while the patient’s degenerative spinal changes may have compromised local immune surveillance. Though *A. vaginalis* virulence factors remain poorly characterized, its pathogenic potential may involve biofilm formation - a trait documented in related anaerobic cocci like *Peptostreptococcus* - which could explain the chronic, localized nature of the infection despite negative cultures. The organism’s low virulence signature (evidenced by mild systemic inflammation despite significant disc destruction) and potential synergistic interactions with undetected co-pathogens represent important areas for future research. These biological considerations reinforce why NTS’s rapid detection capability was critical for diagnosis, while highlighting the need to complement sequencing with studies of microbial pathogenicity in such novel infections.

Opportunistic pathogens usually represented persistent low-invasive infections under certain circumstances, such as compromised immunity. Spondylodiscitis caused by opportunistic pathogens was relatively rare. The reported opportunistic pathogens included *Veillonella parvula* (11), Streptococcus tigurinus (12), *Rhodococcus equi* (13), *Aerococcus urinae* (14), *Parvimonas micra* (15), *Rothia dentocariosa* (16) and *Enterococcus faecalis* (17). This study provides the first documented case of spondylodiscitis caused by *Anaerococcus vaginalis*, thereby contributing to the emerging research domain of spondylodiscitis and enhancing the understanding of both the condition and the pathogen *Anaerococcus vaginalis*.

While NTS proved instrumental in identifying *Anaerococcus vaginalis* in this case, its limitations—such as the inability to determine strain virulence, typing, or drug-resistance genes—warrant careful consideration. The absence of virulence data could impact prognosis assessment, though our patient’s clinical response suggested low virulence. Similarly, while NTS guided our antibiotic selection (switching to metronidazole), the lack of resistance gene detection highlights the potential value of supplementing NTS with targeted PCR or culture-based susceptibility testing in complex cases. To optimize future applications, we propose a multimodal approach combining NTS with clinical correlation, traditional cultures, and targeted assays to balance speed with comprehensive pathogen characterization. Advances in bioinformatics, including expanded resistance databases and standardized protocols, may further enhance NTS’s utility. These strategies would mitigate current limitations while preserving NTS’s advantages in rapid, sensitive pathogen detection for rare infections like *A. vaginalis* spondylodiscitis. Therefore, comprehensive considerations were imperative for specialists to formulate a diagnosis (18).

## 4 Conclusion

This study presented the initial report of spondylodiscitis caused by *Anaerococcus vaginalis*. In instances of infrequent bacterial spondylodiscitis, conventional detection techniques exhibited limited efficacy, however, NTS was an effective strategy to mitigate the incidence of missed diagnosis.

## Data Availability

The original contributions presented in the study are included in the article/supplementary material, further inquiries can be directed to the corresponding author/s.
